# A Rare Case of Descending Colon Metastasis Following Radical Nephroureterectomy for Left Ureteral Carcinoma: A Case Report and Literature Review

**DOI:** 10.3390/curroncol33040235

**Published:** 2026-04-21

**Authors:** Huaiwen Zhang, Heyang Liu, Yousong Luo, Peizhe Li, Lianjun Yang, Jing Shi, Junyao Duan, Yongji Yan

**Affiliations:** 1 Department of Urology, Dongzhimen Hospital, Beijing University of Chinese Medicine, Beijing 100700, China; 2 Department of Pathology, Dongzhimen Hospital, Beijing University of Chinese Medicine, Beijing 100700, China

**Keywords:** upper tract urothelial carcinoma, metastatic urothelial carcinoma, descending colon metastasis, biopsy, gemcitabine-cisplatin chemotherapy, platinum resistance, antibody-drug conjugates

## Abstract

Upper tract urothelial carcinoma (UTUC) is a rare and aggressive malignancy, accounting for only 5–10% of all urothelial carcinomas (UCs). Lung, bone, liver, and distant lymph nodes are common sites of metastasis, while gastrointestinal metastasis is extremely rare. We present a case of a 63-year-old female who developed a descending colon lesion 19 months after left radical nephroureterectomy for high-grade ureteral UC. The diagnosis was established by computed tomography (CT), magnetic resonance imaging (MRI), colonoscopy, and biopsy, which excluded primary colorectal malignancy. First-line therapy consisted of six 21-day cycles of gemcitabine plus cisplatin, followed by two cycles of tislelizumab maintenance immunotherapy. Restaging with contrast-enhanced CT and positron emission tomography/computed tomography (PET/CT) demonstrated disease progression. Despite switching to second-line nab-paclitaxel, the patient rapidly deteriorated from tumor cachexia and ultimately succumbed to septic shock secondary to severe pulmonary infection. This represents the first reported case of descending colon metastasis from primary ureteral UC. It highlights the colon as a potential metastatic site where biopsy is essential for definitive diagnosis. Notably, although the patient initially responded to platinum-based therapy, the subsequent rapid progression underscores the need for vigilant monitoring and timely adjustment of therapeutic strategies in managing such high-risk presentations.

## Simple Summary

Upper tract urothelial carcinoma is a rare and aggressive cancer that typically spreads to the lungs, bones, liver, or distant lymph nodes but rarely to the gastrointestinal tract. This report describes the first known case of such a cancer originating in the left ureter and metastasizing to the descending colon 19 months after surgical removal of the ureter. Despite initial responses to platinum-based chemotherapy, the disease progressed quickly during follow-up immunotherapy, leading to the patient’s death from complications. These findings emphasize that the colon can be an unusual site of spread, highlighting the need for biopsy confirmation to distinguish it from primary colon cancer and for close monitoring to enable timely shifts to advanced therapies like targeted drugs, potentially improving outcomes in similar rare cases.

## 1. Introduction

Upper tract urothelial carcinoma (UTUC) is a relatively rare malignancy, accounting for only 5–10% of all urothelial carcinomas (UCs), with an estimated overall incidence of 1–2 cases per 100,000 persons [1,2]. In Western populations, UTUC is twice as common in men as in women, and tumors occur in the renal pelvis about twice as frequently as in the ureter [3]. Compared to bladder UC, UTUC exhibits more aggressive biological behavior, with nearly 60% of patients presenting with invasive disease at the time of initial diagnosis [4].

The management of UTUC is stratified by risk. While kidney-sparing surgery (KSS) is an option for low-risk disease, radical nephroureterectomy (RNU) remains the standard curative treatment for high-risk patients [5]. Metastasis is frequently observed in advanced UTUC [6], with the lungs, bones, liver, and distant lymph nodes being the most common metastatic sites [7]. In contrast, gastrointestinal involvement, particularly metastasis to the colon, is exceedingly rare, with only sporadic cases reported in the literature.

This report presents the first documented case of descending colon metastasis originating from primary ureteral UC, occurring 19 months after left RNU. This case and the accompanying literature review aim to discuss the diagnostic challenges and therapeutic management associated with this unusual metastatic pattern.

## 2. Case Presentation

A 63-year-old woman presented with a one-month history of urinary frequency, dysuria, gross hematuria, left lumbar pain, and unintentional weight loss of 8 kg. Nineteen months prior to this admission, initial contrast-enhanced computed tomography (CT) revealed a 2.3 × 2.2 cm enhancing soft-tissue mass at the left distal ureter involving the ureterovesical junction. Although adjacent nodular shadows on CT initially raised suspicion for regional lymphadenopathy, a formal regional lymphadenectomy was omitted as intraoperative assessment suggested these were likely reactive inflammatory changes rather than definitive metastases. The patient subsequently underwent a transperitoneal laparoscopic radical nephroureterectomy, during which the ureteral detachment and bladder cuff excision were performed intracorporeally. To prevent tumor cell seeding, strict oncological principles were meticulously observed during the procedure. First, prior to opening the urinary tract, intravesical instillation of epirubicin was performed via the urethral catheter. Second, during the bladder cuff excision, continuous suction was applied to immediately clear any urinary efflux, avoiding spillage into the operative field. Finally, the excised specimen was promptly placed into an impermeable retrieval bag to prevent direct contact with the abdominopelvic cavity. The final pathology confirmed a high-grade UTUC invading the lamina propria with negative surgical margins. Consequently, the definitive clinicopathological stage was pT1NxM0 (0 nodes resected), with no explicitly enlarged lymph nodes identified within the resected renal hilum and perirenal adipose tissue. Following discharge, the patient exhibited poor compliance and did not undergo routine cystoscopy or imaging surveillance according to guideline recommendations.

**Figure 1:**
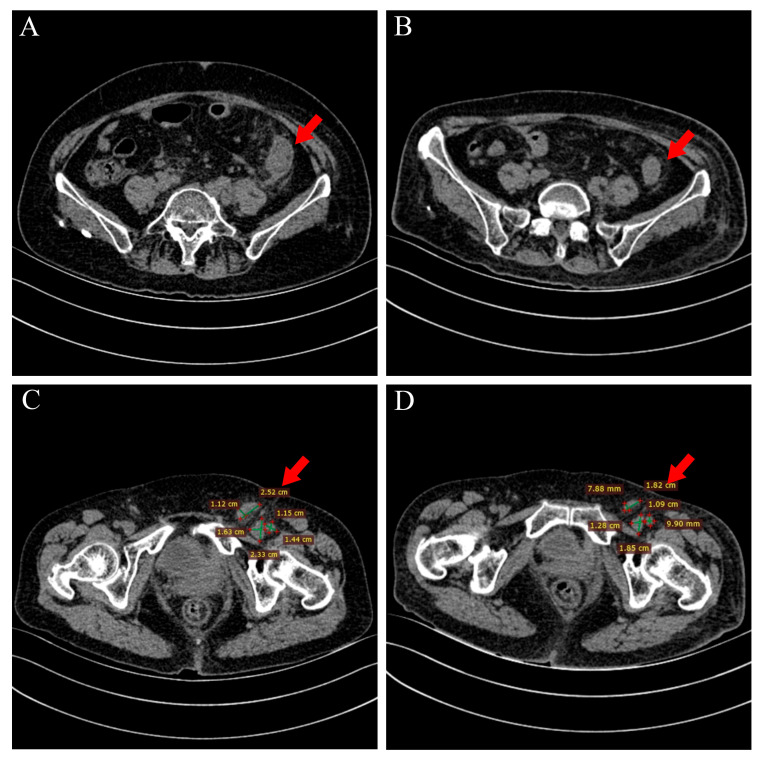
Whole-abdominal non-contrast CT scan. (**A**) Pre-treatment axial image shows circumferential wall thickening of the descending colon and increased pericolonic fat stranding. (**B**) Follow-up axial CT scan at the same level after two cycles of GC regimen shows marked regression of colonic wall thickening. (**C**) Baseline scan shows an enlarged left inguinal lymph node, largest dimension 2.52 × 1.63 cm. (**D**) Post-chemotherapy scan at the same level shows significant lymph node reduction, largest dimension 1.82 × 0.79 cm.

**Figure 2:**
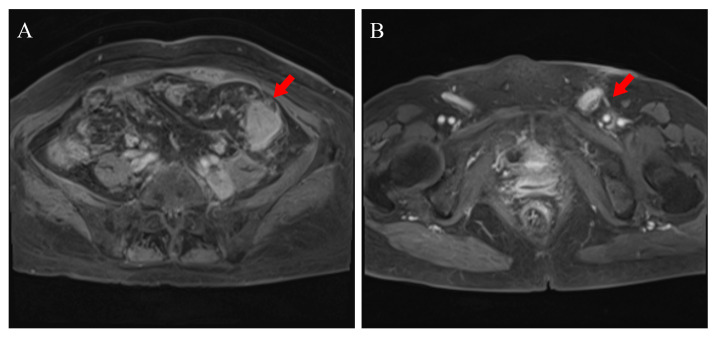
Contrast-enhanced abdominal MRI. (**A**) Axial fat-suppressed T1-weighted image (portal venous phase) shows irregular circumferential thickening and marked enhancement of the descending colon wall. (**B**) Axial fat-suppressed T1-weighted image reveals enlarged, heterogeneously enhancing lymph node clusters in the left inguinal region.

**Figure 3:**
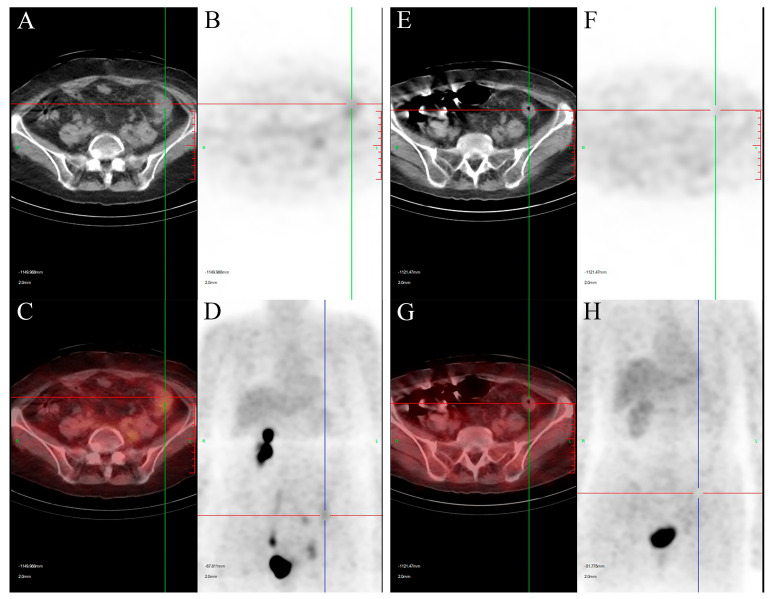
PET/CT evaluation of the descending colon lesion. Left panels (pre-treatment): (**A**) Axial CT shows localized wall thickening of the descending colon with blurred surrounding fat planes. (**B**) Corresponding PET image shows focal abnormal radiotracer uptake in the colonic lesion. (**C**) Fused image confirms colocalization of the hypermetabolic area with the anatomical abnormality. (**D**) Whole-body maximum intensity projection (MIP) image highlights the hypermetabolic descending colon lesion. Right panels (post-treatment): (**E**) Axial CT image shows significant improvement in pericolonic infiltration. (**F**) Axial PET and (**H**) MIP images demonstrate markedly reduced metabolic activity in the colonic lesion. (**G**) Fused image confirms reduced metabolic activity and lesion size.

**Figure 4:**
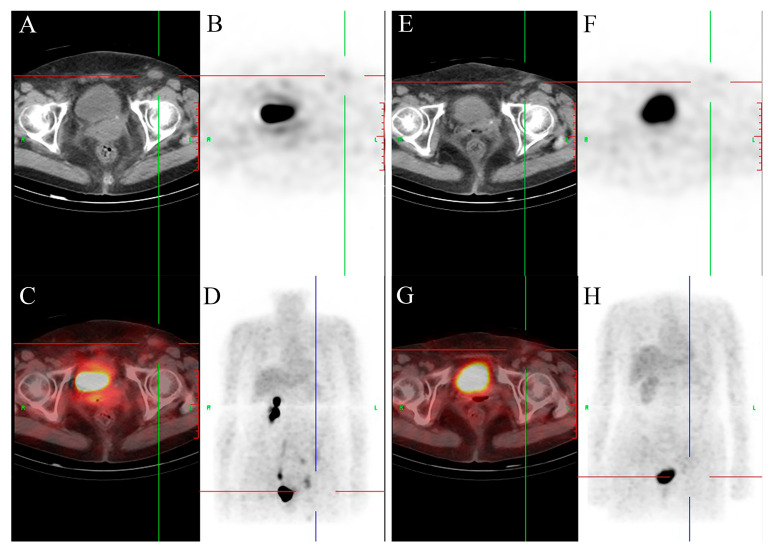
PET/CT evaluation of the left inguinal lymph node. Left panels (pre-treatment): (**A**) Axial CT image shows an enlarged left inguinal lymph node. (**B**) Corresponding PET image shows focal abnormal radiotracer uptake, indicating high metabolic activity. (**C**) Fused image confirms the hypermetabolic node corresponds to the anatomical enlargement. (**D**) Whole-body MIP shows a prominent hypermetabolic focus in the left inguinal region. Right panels (post-treatment): (**E**) Axial CT shows significant reduction in lymph node size. (**F**) PET and (**H**) MIP images show markedly decreased metabolic activity. (**G**) Fused image confirms lymph node shrinkage and metabolic reduction.

Nineteen months later, on admission, she presented with lower abdominal distension, poor appetite, nausea, vomiting, urinary frequency (daytime frequency > 10 times, and nocturia 4–5 times), and loose stools. She denied a family history of malignancy but reported allergies to penicillin and streptomycin. Non-contrast abdominal CT showed increased fat stranding around the descending and sigmoid colon (Figure 1A) and multiple enlarged lymph nodes in the abdominal cavity, retroperitoneum, left pelvis, and left inguinal region (Figure 1C). Contrast-enhanced CT confirmed abnormal enhancement of these lesions, suspicious for malignancy. Pelvic magnetic resonance imaging (MRI) demonstrated irregular circumferential thickening of the left descending colon wall (Figure 2A) with adjacent enlarged lymph nodes (Figure 2B). Whole-body positron emission tomography/computed tomography (PET/CT) revealed focal uptake corresponding to the descending colonic lesion and high-metabolic activity in multiple nodal stations (Figure 3 and Figure 4, left panels).

**Figure 5:**
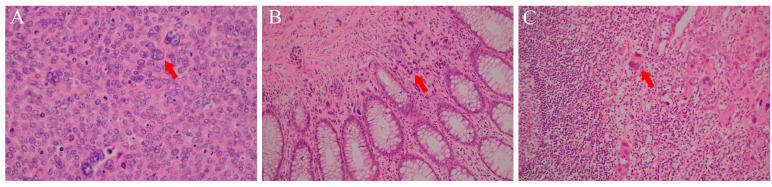
Intraoperative pathology sections were stained with hematoxylin and eosin (HE). The magnifications for (**A**–**C**) were ×400, ×200, and ×200, respectively. (**A**) Bladder mucosal tissue. (**B**) Colonic mucosal tissue. (**C**) Left inguinal lymph node tissue.

**Figure 6:**
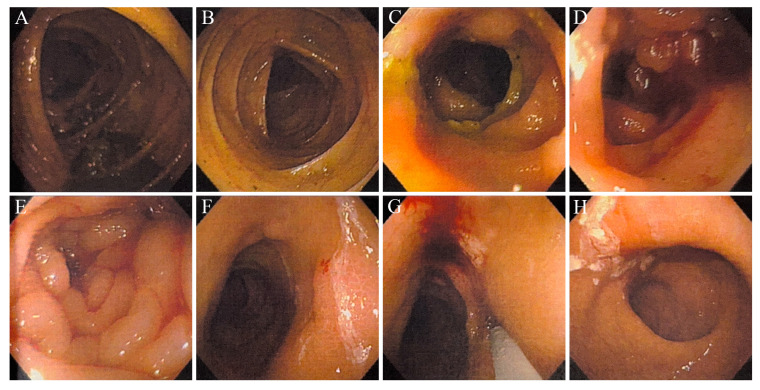
Colonoscopy findings. (**A**,**B**) The ileocecal junction and transverse colon show normal mucosa and vascular patterns. (**C**–**E**) The descending colon (35–45 cm) from the anal verge shows marked edema, mucosal irregularity, and luminal narrowing. (**F**,**G**) The rectosigmoid junction (15 cm from anal verge) shows multiple rough mucosal elevations. (**H**) Rectal mucosa shows leopard-skin-like pigmentation without neoplasia.

Cystoscopy showed follicular changes around the left ureteral orifice. The biopsy confirmed UC in situ with associated adenomatous and papillary cystitis (Figure 5A). Immunohistochemistry (IHC) showed full-thickness, weak to moderate positivity for p53. Colonoscopy identified infiltrating, poorly differentiated carcinoma at two locations: 15 cm and 35–45 cm from the anal verge (Figure 6). A biopsy of the colonic lesions revealed carcinoma (Figure 5B). IHC of the colonic lesions supported a urothelial origin: cytokeratin (CK) (+), p63 (+), chromogranin A (CgA) (−), synaptophysin (Syn) (−), Ki-67 ≈ 70% (hot-spot), and vimentin (Vim) (−). A biopsy of a left inguinal lymph node revealed metastatic poorly differentiated carcinoma, with a micropapillary pattern and tumor emboli (Figure 5C). IHC of the nodal tissue was consistent with urothelial origin: p63 (+), CK7 (3+), CK20 (−), Villin (−). Taken together with the prior history of ureteral UC, these findings supported a diagnosis of metastatic urothelial carcinoma (mUC) (stage IV) with descending colon involvement.

Given the chronological context of her diagnosis in 2021, the patient was initiated on a gemcitabine–cisplatin (GC) regimen, which was the established standard-of-care first-line systemic therapy at that time. The 21-day cycle regimen consisted of gemcitabine (1000 mg/m^2^ on days 1 and 8) plus cisplatin (75 mg/m^2^ on day 2). Following the completion of the first two cycles, non-contrast CT and PET/CT demonstrated marked regression of pericolonic fat stranding and substantial reduction in the size and metabolic activity of metastatic lymph nodes and the colonic lesion (Figure 1B,D, Figure 3 and Figure 4, right panels). The patient completed a total of six cycles of this GC regimen, which was well-tolerated except for transient vomiting during the fourth cycle, with no evidence of disease progression at that time. Subsequently, immunotherapy maintenance was initiated with tislelizumab (200 mg intravenously every 3 weeks). After two cycles, a follow-up PET/CT revealed multiple new pulmonary nodules with mildly increased metabolism, indicating disease progression.

The therapeutic regimen was switched to second-line nab-paclitaxel (370 mg). However, during this period, the patient experienced rapid clinical deterioration characterized by tumor cachexia. She suffered a fall with subsequent impaired consciousness and developed aspiration pneumonia. Despite intensive supportive care, she succumbed to septic shock secondary to severe pulmonary infection.

## 3. Discussion

UTUC carries a significant risk of recurrence after RNU, with approximately 20–30% of patients developing distant metastases within three years [8,9]. The most frequent sites of metastasis are lungs, bones, liver, and distant lymph nodes, while gastrointestinal involvement is exceedingly rare [7]. Although sporadic reports have described atypical metastatic sites like the ileum [10], testes [11], skin [12], heart [13], skeletal muscle [13,14] and choroid [15] (summarized in Table 1), distant metastasis to the colon is distinctly uncommon.

Critically, this pattern must be distinguished from direct contiguous invasion. Unlike the case described by Yang et al. [12], where colonic involvement resulted from local extension of a renal pelvic tumor, our patient exhibited an isolated lesion in a non-adjacent segment of the descending colon. As noted in the literature, atypical recurrence patterns—such as peritoneal implantation due to pneumoperitoneum aerosolization or intraoperative spillage—have been reported following minimally invasive surgeries [2,16], requiring us to carefully evaluate the possibility of iatrogenic tumor dissemination. However, as detailed previously, rigorous preventative measures—including preoperative intravesical instillation, continuous suction to preclude urinary spillage, and immediate specimen bagging—were strictly implemented during the initial laparoscopic procedure. Given these meticulous oncological precautions, the probability of iatrogenic tumor implantation was minimized. Therefore, while a theoretical risk cannot be completely excluded, this clinical presentation strongly supports a true distant metastasis. To the best of our knowledge, this is the first reported case of a descending colon metastasis originating from a primary ureteral UC.

Furthermore, the specific pattern of extraurothelial spread is closely related to underlying tumor biology. Recent literature [17,18] emphasizes that metastatic tropism and disease aggressiveness in UTUC can vary significantly depending on histological subtypes. While our patient’s primary pathology indicated high-grade urothelial carcinoma without divergent differentiation, high-grade UTUC itself is inherently aggressive and possesses substantial metastatic potential. This inherent biological aggressiveness strongly predisposes the tumor to atypical dissemination, offering a compelling explanation for the isolated colonic metastasis observed in our patient.

The diagnosis in this case was particularly challenging because the patient presented with nonspecific gastrointestinal symptoms (weight loss and loose stools) and the non-adjacent anatomical location of the colonic lesions, which could easily have been misattributed to a primary gastrointestinal malignancy. While imaging and endoscopy indicated malignant infiltration, they could not determine the origin. Therefore, endoscopic biopsies were performed for definitive pathological evaluation. The colonic biopsy showed positivity for CK and p63 (a highly specific marker for UC) [19,20], and negativity for neuroendocrine (CgA, Syn) and mesenchymal (Vim) markers. This profile strongly supported a urothelial origin [21,22,23,24,25,26,27]. Although CK7/CK20 were not tested on the colon specimen, the congruent IHC result from the inguinal lymph node metastasis (CK7+/CK20−/p63+) provided corroborating evidence for mUC [26,27]. This case underscores the necessity of obtaining definitive histological evidence through biopsy when evaluating atypical colonic lesions in patients with prior UTUC, rather than relying solely on imaging findings.

In accordance with the European Association of Urology (EAU) Guidelines, cisplatin-based combination chemotherapy has long been established as the standard first-line treatment for patients with locally advanced or metastatic urothelial carcinoma (la/mUC) [28,29,30]. Consistent with these recommendations, our patient received first-line systemic therapy with GC regimen. Initially, the tumor demonstrated platinum sensitivity, as evidenced by the marked regression of both the colonic lesion and metastatic lymph nodes after two cycles. Following the completion of six chemotherapy cycles, the patient underwent maintenance immunotherapy with tislelizumab. However, this response was short-lived. Disease progression emerged rapidly during the maintenance phase (after only two cycles), with new pulmonary lesions appearing. Such a swift transition from initial sensitivity to acquired resistance suggests the high biological heterogeneity and aggressive nature often observed in advanced UTUC. The mechanisms of platinum resistance in urothelial carcinoma are multifaceted and complex. Available evidence indicates that drug resistance may involve reduced drug accumulation mediated by drug transporters, enhanced DNA repair systems, and suppression of apoptotic signaling pathways [31,32]. Faced with this aggressive progression, the treatment strategy was escalated to a second-line nab-paclitaxel. Unfortunately, the disease proved refractory even to this second-line therapy. The patient experienced continuous deterioration and ultimately succumbed to the disease. The rapid progression despite sequential administration of standard chemotherapy and immunotherapy highlights a significant clinical challenge in managing aggressive metastatic urinary tract urothelial carcinoma (mUTUC). This rapid resistance to conventional systemic therapies underscores the limited empirical treatment options and overall poor prognosis for patients with highly aggressive, pre-treated metastatic urothelial carcinoma [33].

For such patients, antibody-drug conjugates (ADCs) and targeted therapies emerge as critical later-line strategies. Research confirms that enfortumab vedotin (EV), an ADC targeting Nectin-4, significantly extends overall survival (OS) in patients who have undergone platinum-based chemotherapy and immunotherapy compared to chemotherapy alone (median OS: 12.88 months vs. 8.97 months) [34]. Notably, currently, EV combined with pembrolizumab as a first-line regimen nearly doubled patient survival compared to chemotherapy (median OS: 31.5 months vs. 16.1 months), establishing it as a preferred option in European and American guidelines [35,36,37]. Other promising ADCs include sacituzumab govitecan (SG; TROP-2-targeted), trastuzumab deruxtecan (T-DXd; HER2-targeted), and disitamab vedotin (RC48; HER2-targeted), all demonstrating notable efficacy in biomarker-selected populations [38,39,40,41]. Beyond ADCs, targeting specific molecular alterations is another key approach. The incidence of FGFR3 genetic alterations is particularly high in UTUC (up to 43%), significantly exceeding that in bladder UC [42]. For patients with such alterations, erdafitinib, an oral FGFR inhibitor, significantly prolonged OS compared to chemotherapy (median OS: 23.3 months vs. 11.3 months) [43].

In our case, due to financial toxicity and limited drug accessibility at the time, the patient could not obtain ADC therapy. Instead, she received a more affordable immunotherapy option (tislelizumab) and chemotherapy, which provided a more viable economic alternative. However, the rapid progression observed with this empirical approach underscores the urgent need for personalized strategies. Therefore, for cases of resistant or rare mUTUC, systematic biomarker testing (e.g., FGFR3, HER2) may be considered upon confirmation of metastatic disease. This provides critical evidence for subsequent precision selection of ADCs or targeted therapies.

## 4. Conclusions

In conclusion, this case underscores the importance of considering the possibility of UTUC metastasis when evaluating new gastrointestinal lesions in patients with a prior history of UTUC. Definitive biopsy remains essential to exclude primary colorectal cancer and confirm metastatic disease. The rapid progression observed after initial platinum-based response highlights the aggressive behavior of such metastases. Our findings suggest that early molecular profiling and vigilant monitoring are critical for the timely adaptation of therapeutic strategies, such as ADCs or targeted therapies, in these high-risk clinical scenarios.

## Figures and Tables

**Table 1:** Reported cases of rare metastatic sites in upper tract urothelial carcinoma.

No.	Author (Year)	Age/Sex	Primary Tumor Site	Rare Metastatic Site	Treatment (Primary)	Diagnosis Modality	Treatment (Metastasis)	Outcome
1	Ren et al. (2021) [10]	63/F	Right Ureter	Ileum	Right RNU	CT, Biopsy	Tislelizumab + Gem/Carbo	Died (4 mo post-Met)
2	Wu et al. (2020) [11]	57/M	Left renal pelvis	Testis	Left RNU, Gem/Neda	US, Biopsy	Radical Orchiectomy	Died (1 mo post-Met)
3	Yang et al. (2024) [12]	63/M	Left renal pelvis	Skin (metastasis) and descending colon (invasion)	Left RNU, left hemicolectomy, Gem/Carbo	Biopsy	Excision, Pembrolizumab	NED (15 mo post-RNU)
4	Chan et al. (2024) [13]	58/M	Left Renal Pelvis	Heart, skeletal muscle	Left RNU	CT, Enhanced CT	Pembrolizumab (2 yr)	CR (post 2 yr Tx)
5	Friesen et al. (2024) [14]	66/M	Right Renal Pelvis	Skeletal muscle	Untreated	FDG-PET, Biopsy	GC	Died (4 wk post-Mets)
6	Che et al. (2025) [15]	51/F	Left Renal Pelvis	Choroid	Untreated	Fundoscopy, ICG-A, Doppler US, OCT, MRI	Untreated	Died (2 wk post-Mets)

Abbreviations: F = Female; M = Male; RNU = radical nephroureterectomy; GC = Gemcitabine + Cisplatin; Gem/Neda = Gemcitabine + Nedaplatin; Gem/Carbo = Gemcitabine + Carboplatin; CT = computed tomography; US = ultrasound; FDG-PET = Fluorodeoxyglucose Positron Emission Tomography; ICG-A = indocyanine green angiography; OCT = Optical Coherence Tomography; MRI = magnetic resonance imaging; yr = years; mo = months; wk = weeks; NED = no evidence of disease; CR = complete response. Note: Timeframes in the Outcome column are calculated from different starting points as reported in the original studies: post-Mets: from the diagnosis of metastasis; post-RNU: from the primary radical nephroureterectomy; post-Tx: after completion of immunotherapy treatment.

## Data Availability

Data are contained within the article. Further inquiries can be directed to the corresponding author. The raw clinical records cannot be shared publicly due to patient privacy and confidentiality requirements.

## Abbreviations

The following abbreviations are used in this manuscript:

UTUC	Upper urinary tract urothelial carcinoma
UC	Urothelial carcinoma
RNU	Radical nephroureterectomy
CT	Computed tomography
MRI	Magnetic resonance imaging
GC	Gemcitabine plus cisplatin
PET/CT	Positron emission tomography/computed tomography
KSS	Kidney-sparing surgery
IHC	Immunohistochemistry
CK	Cytokeratin
CgA	Chromogranin A
Syn	Synaptophysin
Vim	Vimentin
MIP	Maximum intensity projection
EAU	European Association of Urology
la/mUC	Locally advanced or metastatic urothelial carcinoma
mUTUC	Metastatic urinary tract urothelial carcinoma
ADC	Antibody-drug conjugate
EV	Enfortumab vedotin
OS	Overall survival
SG	Sacituzumab govitecan
T-DXd	Trastuzumab deruxtecan
RC48	Disitamab vedotin
